# Genetically engineered *MAPT* 10+16 mutation causes pathophysiological excitability of human iPSC-derived neurons related to 4R tau-induced dementia

**DOI:** 10.1038/s41419-021-04007-w

**Published:** 2021-07-17

**Authors:** Olga Kopach, Noemí Esteras, Selina Wray, Andrey Y. Abramov, Dmitri A. Rusakov

**Affiliations:** 1 Department of Clinical and Experimental Epilepsy, UCL Queen Square Institute of Neurology, London, UK; 2 Department of Clinical and Movement Neurosciences, UCL Queen Square Institute of Neurology, London, UK; 3 Department of Neurodegenerative Disease, UCL Queen Square Institute of Neurology, London, UK

**Keywords:** Mechanisms of disease, Cellular neuroscience, Dementia

## Abstract

Human iPSC lines represent a powerful translational model of tauopathies. We have recently described a pathophysiological phenotype of neuronal excitability of human cells derived from the patients with familial frontotemporal dementia and parkinsonism (FTDP-17) caused by the *MAPT* 10+16 splice-site mutation. This mutation leads to the increased splicing of 4R tau isoforms. However, the role of different isoforms of tau protein in initiating neuronal dementia-related dysfunction, and the causality between the *MAPT* 10+16 mutation and altered neuronal activity have remained unclear. Here, we employed genetically engineered cells, in which the IVS10+16 mutation was introduced into healthy donor iPSCs to increase the expression of 4R tau isoform in exon 10, aiming to explore key physiological traits of iPSC-derived *MAPT* IVS10+16 neurons using patch-clamp electrophysiology and multiphoton fluorescent imaging techniques. We found that during late in vitro neurogenesis (from ~180 to 230 days) iPSC-derived cortical neurons of the control group (parental wild-type tau) exhibited membrane properties compatible with “mature” neurons. In contrast, *MAPT* IVS10+16 neurons displayed impaired excitability, as reflected by a depolarized resting membrane potential, an increased input resistance, and reduced voltage-gated Na^+^- and K^+^-channel-mediated currents. The mutation changed the channel properties of fast-inactivating Na_v_ and decreased the Na_v_1.6 protein level. *MAPT* IVS10+16 neurons exhibited reduced firing accompanied by a changed action potential waveform and severely disturbed intracellular Ca^2+^ dynamics, both in the soma and dendrites, upon neuronal depolarization. These results unveil a causal link between the *MAPT* 10+16 mutation, hence overproduction of 4R tau, and a dysfunction of human cells, identifying a biophysical basis of changed neuronal activity in 4R tau-triggered dementia. Our study lends further support to using iPSC lines as a suitable platform for modelling tau-induced human neuropathology in vitro.

## Introduction

The deposition of abnormal tau protein is a hallmark for a large group of human cognitive disorders (tauopathies), which includes Alzheimer’s disease [1, 2], several forms of parkinsonism [3] or frontotemporal lobar degeneration (FTLD)—such as corticobasal degeneration, progressive supranuclear palsy, inherited frontotemporal dementia and parkinsonism linked to chromosome 17 (FTDP-17)—among others [4–6]. The primary underlying molecular mechanism, as established to date, includes genetically triggered self-aggregation of tau protein [7] followed by conformational changes in the microtubule dynamics [8, 9]. The latter can provoke neurodegeneration due to induced neuronal cell death—directly or via calcium-induced excitotoxicity [10–12]—and ultimately a cognitive decline in patients with tauopathy [13].

The microtubule-associated protein tau assembles into multiple, highly versatile types of filaments. The human tau is encoded by the *MAPT* gene on chromosome 17, and alternative splicing of exons 2, 3, and 10 of the gene 17q21 leads to the expression of six tau isoforms in the adult human brain. The structure of tau isoforms differs substantially in their tubulin-binding domains, varying between three-repeat (3R) or four-repeat (4R) tau [1]. The inclusion of exon 10 leads to the expression of 4R tau, while its exclusion generates the 3R isoforms. Cumulative evidence indicates that correct splicing (balanced 3R/4R ratio) is required for normal neuronal function: several *MAPT* mutations causing overproduction of 4R tau (inclusion of exon 10) have effectively triggered neurodegeneration linked to dementia [4–6]. Although immense progress has been made over the past decade in our understanding of the molecular biology of tau protein, the exact mechanism(s) by which various tau isoforms affect neuronal activity and thus initiate neuronal dysfunction remain largely unclear.

Recent advances in generating neural cells using induced pluripotent stem cell (iPSC) technologies have provided a well-established platform for modelling neuropathology in vitro aimed at probing live human cells directly. Human iPSC-derived neurons recapitulate the developmental splicing of tau isoforms (increased 4R tau) as in the adult human brain and replicate the mutations linked to tau pathogenesis [14–16]. We have recently confirmed the functional reliability of human iPSC-derived cortical neurons at extended neurogenesis and have described the phenotypes of pathophysiological excitability [17] and mitochondrial dysfunction [18] of human cells derived from patients with FTDP-17 which is related to the intronic *MAPT* 10+16 mutation. To experimentally promote the inclusion of exon 10, hence overexpression of 4R tau isoform, this mutation was genetically engineered in healthy donor iPSC lines [19]. The subsequent characterization of genetically engineered neurons has confirmed the increased expression of 4R tau protein and its hyperphosphorylation at various phosphorylation sites. In the present study, we ask whether and how the increased splicing of 4R tau isoform relates to neuronal dysfunction, by implementing and exploring this recently established isogenic model of pathogenic 4R tau. Combining single-cell electrophysiology with multiplexed two-photon excitation imaging reveals a causal link between overexpression of 4R tau and pathophysiological excitability of human cells.

## Materials and methods

### Genetically engineered human iPSC lines

This study included two human iPSC lines: one is a genetically engineered *MAPT* IVS10+16 line and another is the isogenic control (wild type at the tau locus, wt tau) line. Both generation and characterization of these two lines were described in detail previously [19]. In brief, the FTDP-17-associated *MAPT* mono-allelic IVS10+16 (+/−) mutation was introduced into a healthy donor line using a zinc finger nuclease technology to increase the inclusion of exon 10. This effectively led to an increased expression of the 4R tau protein [19]. Both gene-edited and isogenic control (wt tau) lines were generated from the same parental line (iPSC0028; Sigma-Aldrich) and are available via the EBISC repository (SIGi001-A-13 and SIGi001-A-1, respectively).

IPSC lines were expanded and maintained as we described in detail previously [16–18]. Briefly, differentiation of the pluripotent stem cells into cortical neurons was performed via dual SMAD inhibition for 10 days followed by extended in vitro neurogenesis. At 46 days after induction, cells were plated on polyornithine/laminin-coated glass coverslips and maintained until use. A neural maintenance medium was a mixture of N2 and B27 media [20], which was changed twice a week. For each group, three different inductions were examined. At least six neuronal cultures (independent preparations) were tested per group. Patch-clamp recordings were carried out at the developmental stages of ~184–189 days in vitro (DIV), and then at ~230 DIV; multiphoton time-lapse imaging recordings were performed at ~210 DIV. All experiments for the *MAPT* IVS10+16 group were performed in conjunction with the age-matched parental isogenic control (wt tau) group. Some patch-clamp experiments were performed by the experimenter in a blind to experimental group manner. The sample size was chosen based on the previous studies [17, 21].

### Immunocytochemistry

Immunocytochemistry was performed using an immunostaining protocol as described previously [17, 21]. The primary antibodies were anti-β-tubulin III and anti-GFAP (1:200; Abcam, UK) incubated with the cells overnight at 4 °C. Hoechst 33342 (10 μM) was used to stain nuclei. Images were acquired as Z-stacks (typically of ~10-μm focal depth) using a Zeiss 710 VIS CLMS confocal microscope. For the quantification of immunofluorescence, Z-stack images were analysed with freely available ImageJ software (NIH, Bethesda, USA). For this, images were processed in 2D mode to separate the GFAP fluorescent signal (green channel) and β-tubulin III immunofluorescence (red channel), the total area of each signal was quantified using an ImageJ plugin for particles/area analysis and normalized to the cell density (nuclei staining). Six neuronal cultures were examined *per* group.

### Electrophysiology

**Fig. 1:**
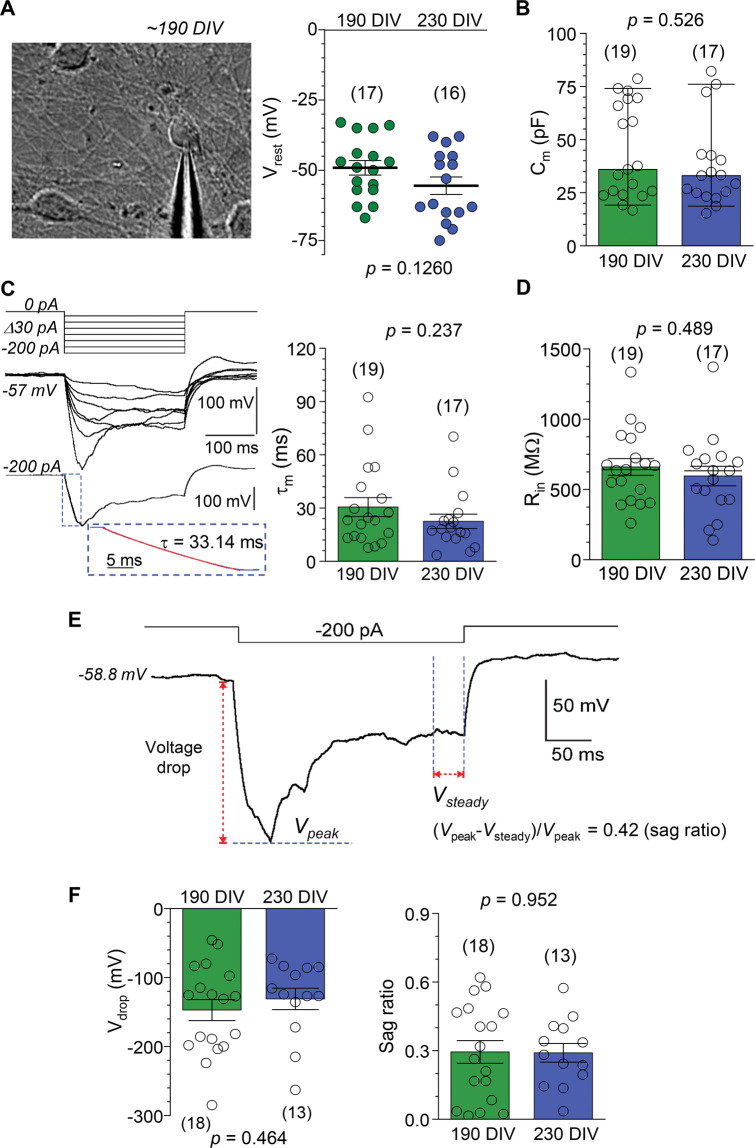
Electrophysiological properties of human iPSC-derived neurons with wild-type tau background even out at late neurogenesis. **A** Left, DIC image of human iPSC-derived neuronal culture at ~190 DIV showing a patched neuron. Right, statistics of the resting membrane potential (*V*_rest_) of iPSC-derived neurons in control (wild type, wt, tau) line at ~190 and 220 DIV. The two-tailed unpaired *t*-test indicated. **B** Statistics of the capacitance (*C*_m_) of iPSC-derived neurons in the control (wt tau) group at different time points. Boxes show median values. Nonparametric Mann–Whitney test indicated. **C** Left, example of neuron responses (current mode) to a series of hyperpolarizing currents (top); lower panel shows how the membrane constant (τ_m_) was measured. Dotted box, the area for calculating τ_m_ (red, linear fit). Right, statistics of the τ_m_ value in wt tau neurons at different time points. The two-tailed unpaired *t*-test indicated. **D** Same as in (**B**), but for the input resistance (*R*_in_). The two-tailed unpaired *t*-test indicated. **E** Representative recording of changes in membrane potential of an iPSC-derived neuron in response to a hyperpolarizing current (indicated on the top) for the calculation of the voltage drop (*V*_drop_) and the sag ratio. **F** Statistical summary for the *V*_drop_ (left) and the sag ratio (right) of control (wt tau) neurons at different time points. The two-tailed unpaired *t*-test indicated. All data are mean with s.e.m, unless indicated. The number of tested cells shown.

Patch-clamp recordings (whole-cell mode) were made from iPSC-derived neurons (Fig. 1A) using a Multipatch 700B amplifier controlled by pClamp 10.2 software package (Molecular Devices, USA). For the recordings, a neuronal culture on a glass coverslip was placed in a recording chamber mounted on the stage of an Olympus BX51WI upright microscope (Olympus, Japan). The cells were continuously superfused with a bicarbonate-buffered solution containing (in mM) 126 NaCl, 3 KCl, 2 MgSO_4_, 2 CaCl_2_, 26 NaHCO_3_, 1.25 NaH_2_PO_4_, 10 D-glucose, which was continuously bubbled with 95% O_2_ and 5% CO_2_ to maintain pH 7.4 (osmolarity 300–310 mOsmol). Recordings were performed at 30–32 °C using glass electrodes with the resistance of 2.5–5 MΩ, filled with an intracellular solution containing (in mM) 126 K-gluconate, 10 HEPES, 4 KCl, 4 MgCl_2_, 2 BAPTA, 4 Mg-ATP, 0.4 GTP-Na (pH adjusted to 7.2 with KOH, osmolarity ~290 mOsmol). In the whole-cell (immediately after membrane breakthrough), iPSC-derived neurons were recorded for the resting membrane potential (*V*_rest_), membrane capacitance (*C*_m_), the membrane time constant (τ_m_), and input resistance (*R*_in_), measured from the hyperpolarizing squire current pulse steps in current mode (Fig. 1C).

To assess the firing activity of iPSC-derived neurons, a series of sub- and supra-threshold rectangular current pulses of 250 ms duration were applied, with a stepwise-increased stimulus intensity (an increment of 20–40 pA), to elicit neuronal firing. The *V*_rest_ was set at −60 mV to −70 mV, by injecting a hyperpolarizing bias current where required.

To obtain the current–voltage (*I–V)* relationship for the Na^+^-channel (Na_v_) and K^+^-channel (Kv)-mediated currents recorded from iPSC-derived neurons, the voltage-clamp protocol was used, which consisted of a series of voltage steps (100–250 ms duration) applied from –90 mV with an increment of 5 mV or 10 mV, as in our earlier study [17]. Capacitance was compensated prior to test recordings.

### Electrophysiological data analysis

The analysis of the action potential (AP) waveform was performed for the first AP only. The parameters of individual APs were: the spike amplitude (measured from the threshold to the peak), the threshold value, and the spike width (duration at half-maximal amplitude), as we described previously [21].

The hyperpolarization-activated cation current (*I*_h_) was calculated as the voltage drop (*V*_drop_) and the sag ratio in cell responses to −200 pA or −170 pA hyperpolarizing pulse injection. The *V*_drop_ was measured as the difference between the voltage peak during hyperpolarizing current and *V*_rest_; the sag ratio was calculated using the equation (*V*_peak_ –*V*_steady_)/*V*_peak_ (Fig. 1E). For the analysis of Na^+^ current (*I*_Na_), the data were leak-corrected using a standard protocol in Clampfit 10.3 software (Molecular Devices). Conductance was determined by dividing the *I*_Na_ value by the membrane potential after subtraction of the experimentally observed reversal potential. Conductance values for K^+^ current (*I*_K_) were obtained by dividing the current amplitude by membrane potential minus K^+^-electrochemical driving force. Normalised conductance (*G*/*G*_max_) was plotted against voltage and fitted by a single Boltzmann function using Origin Pro software (OriginLab, USA).

Persistent inward currents were measured at the end of a 100-ms step pulse, for each membrane voltages, from –50 mV to –10 mV. A relative fraction of persistent current was obtained by dividing the amplitude of inward current (remaining at the end of the 100-ms voltage step) at tested voltage by the peak persistent current for each cell.

### Two-photon excitation (2PE) fluorescent imaging

Cells were bolus loaded with a cell-permeable Ca^2+^ indicator Oregon Green BAPTA-1 (OGB-1 AM; 5 μM, Invitrogen) by incubation for 30 m at 37 °C. After loading, cells were washed for approximately 30 m for de-etherification of the dye. For time-lapse imaging of the OGB-1 signal neuronal cultures were transferred into the bicarbonate-based Rg solution (the same composition as above, continuously saturated with 95% O_2_ and 5% CO_2_) and placed in a recording chamber mounted on the stage of an Olympus BX51WI upright microscope (Olympus, Japan). Imaging was carried out using an Olympus FV1000 imaging system optically linked a Ti:Sapphire MaiTai femtosecond-pulse laser (SpectraPhysics-Newport) at λ^2P^ex = 800 nm (OGB-1 optimum), with appropriate emission filters, as detailed earlier [22–24]. The two-photon excitation regime, which collects fluorescence only within a thin (~1 μm) focal plane, was used to minimize contaminating fluorescence in culture [24, 25], such as autofluorescence arising from the protein-enriched layers on the bottom of coverslips that support human cells over a long-term iPSC culture maintenance. The focal plane within individual cells was held unchanged throughout the recording. For the time-lapse imaging of the OGB-1 signal in iPSC-derived neurons (before and following neuronal depolarization), images were collected in a stream acquisition mode. Images were acquired as 512 × 512-pixel frame scans [24]. To avoid phototoxic damage to the cells during scanning, the laser power was always kept at its reasonable minimum.

Neuronal depolarization was elicited pharmacologically, using a brief, local application of high potassium (50 mM) solution (the same composition as above apart from substituting 50 mM of NaCl with 50 mM of KCl). To enable a brief, localised application to the cells, we applied K^+^ solution via a fabricated glass micropipette (∼1 μm diameter of the tip) connected to a PDES-02DX pneumatic microinjector (npi electronic GmbH). A fluorescent tracer Alexa Fluor-594 (AF, 100 μM) was added into the pipette to visualize the area of the puff spread and to provide the time-stamp of depolarizing stimulus [22, 24, 26]. Changes in the intracellular Ca^2+^ level were expressed as the changes in OGB-1 fluorescence over baseline (Δ*F*/*F*_0_).

### Western blot

Cell cultures were washed with PBS and lysed in an ice-cold RIPA lysis buffer supplemented with protease and phosphatase inhibitors (ThermoFisher, Paisley, UK). Samples were snap-frozen and used as described in detail in our earlier study [17]. Briefly, cultured cells were centrifuged (14000 rpm) and the protein content was determined using the Pierce™ BCA protein assay (ThermoFisher, UK). Protein was fractionated on SDS polyacrylamide gel (ThermoFisher, UK) and transferred to a PVDF membrane (Bio-Rad, Richmond, CA). The primary antibodies were: Na_v_1.6 (1:500, Alomone Labs, Jerusalem, Israel, ASC-009) and β-tubulin III (1:5000, Cell Signaling Technologies, MA, USA, MAB1195) diluted in 5% BSA and incubated overnight. After incubation with the secondary antibodies (for 1 h), the luminol-based Pierce™ ECL Western Blotting Substrate (ThermoFisher Scientific) was used to detect HRP activity. Protein band densities were quantified using ImageJ software (NIH, Maryland, USA) and normalized to the control.

### Statistical analysis

All experimental datasets were tested for normality using the Shapiro–Wilk test. The datasets with normality rejected were presented as median values, and the nonparametric Mann–Whitney test was in this case used for hypothesis testing. For paired comparisons, the nonparametric paired-sample Wilcoxon signed rank test was used. Data are presented as mean ± s.e.m. if distributed normally, with n referring to the number of cells analysed in all cases. For determining the statistical differences between experimental groups, one-way analysis of variance (ANOVA), with an estimate of variation within each group, or the two-tailed unpaired t-test was used as appropriate. *P* < 0.05 was considered as a statistically significant difference between the groups for either test used.

## Results

### Human iPSC-derived neurons display “mature” electrophysiological properties over an extended period of neurogenesis

Human iPSC-derived cortical neurons acquire “mature” membrane properties and intrinsic excitability by the end of ∼150 days of neurogenesis, including the *V*_rest_ and physiological capacity to fire APs [17]. Therefore, we first aimed to validate the maturation of electrophysiological cell properties in the isogenic control line (wt tau), at different time points of late neurogenesis, to rule out possible delays in neuronal maturation. Patch-clamp recordings were performed in control iPSC-derived neurons at ~190 DIV and at ~230 DIV.

In whole-cell configuration, iPSC-derived neurons displayed a prominently negative *V*_rest_, across the time points tested (−49.1 ± 2.6 mV, *n* = 17 at ~190 DIV and −55.5 ± 3.1 mV, *n* = 16 at ~230 DIV, *p* = 0.126; Fig. 1A). The capacitance (*C*_m_) of iPSC-derived neurons was also stable in isogenic control cultures (median value, 44.3 pF, *n* = 19 at ~190 DIV and 37.8 pF, *n* = 17 at ~230 DIV, *p* = 0.526; Mann–Whitney test; Fig. 1B). So were the other passive membrane properties, time constant (τ_m_), and input resistance (*R*_in_): τ_m_ was 30.5 ± 5.3 ms (*n* = 19) at ~190 DIV and 22.5 ± 4.1 ms (*n* = 17) at ~230 DIV (*p* = 0.237; Fig. 1C), and *R*_in_ was 659.6 ± 59.8 MΩ (*n* = 19) and 595.5 ± 69.4 MΩ (*n* = 17), respectively (*p* = 0.489; Fig. 1D). We also measured the cell response to a hyperpolarizing current, which reflects functional expression of nonselective voltage-gated HCN channels activated by cell hyperpolarization. HCN channels are widely expressed in cortical neurons, with particularly high channel expression levels in human pyramidal neurons, across all cortical layers [27], and reportedly play a fundamental role in the integration of synaptic information for memory formation [28]. The parameters of hyperpolarization-activated cation current (*I*_h_)—the *V*_drop_ and the sag ratio (Fig. 1E)—remained unchanged at the time points tested (*V*_drop_: –147.2 ± 15.2 mV, *n* = 18 at ~190 DIV and –131.0 ± 15.5 mV, *n* = 13 at ~230 DIV, *p* = 0.464; the sag ratio: 0.29 ± 0.05, *n* = 18 and 0.29 ± 0.04, *n* = 13, *p* = 0.952, respectively; Fig. 1F).

These data show that electrophysiological parameters of human cells in isogenic cultures with the wt tau background at ~190 DIV or 230 DIV were similar to those of the control iPSC-derived neurons established in our recent study at an earlier time-point, ∼150 DIV [17]. Taken together, these observations indicate that maturation of electrophysiological properties of generated neurons levels out over the extended neurogenesis, between ∼150 and 190 DIV. Therefore, the datasets at ~190 DIV and 230 DIV were subsequently pooled for quantitative comparisons with the genetically engendered *MAPT* IVS10+16 neurons, as described below.

### Genetically engendered *MAPT* IVS10+16 neurons exhibit changed membrane properties

**Fig. 2:**
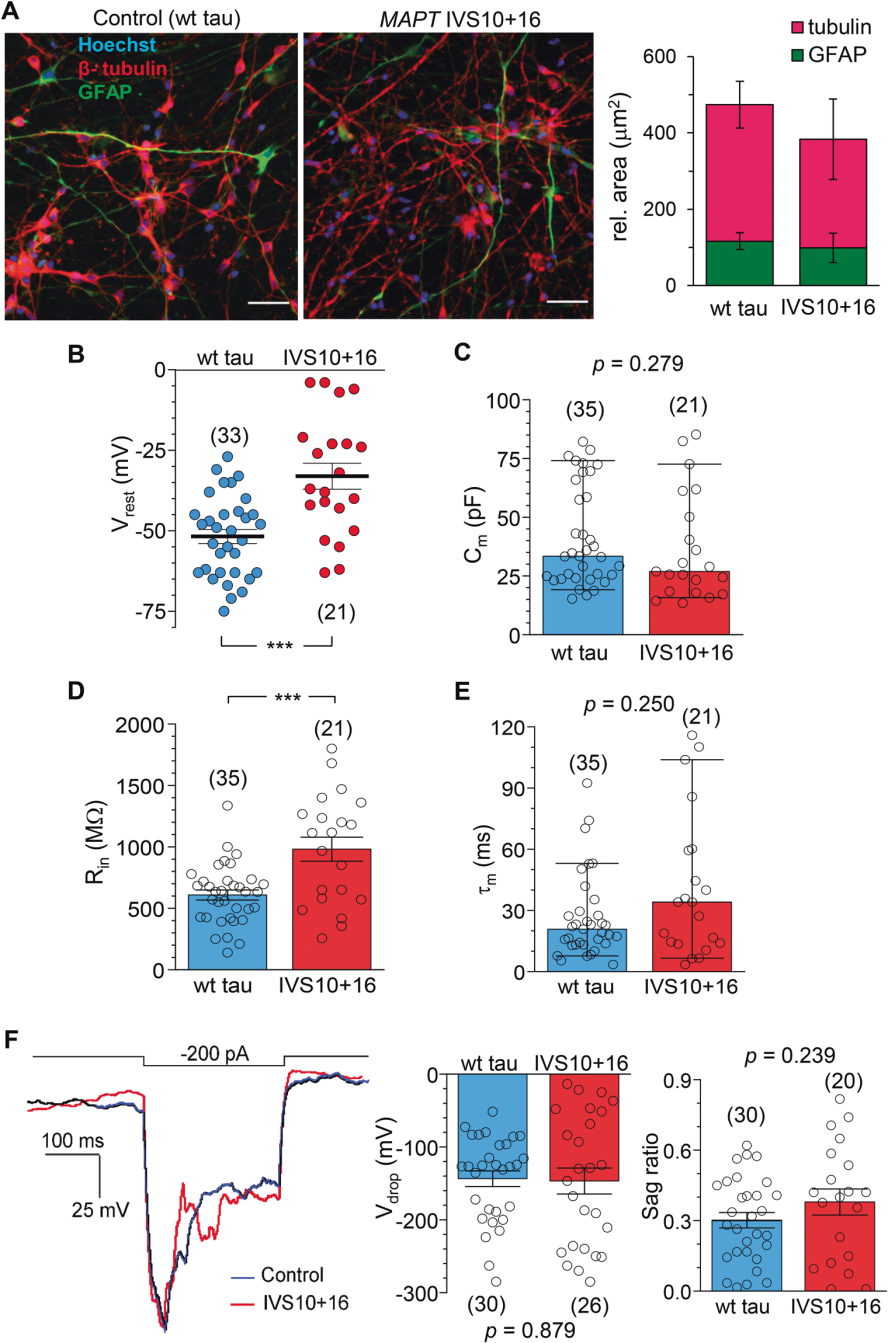
*MAPT* IVS10+16 neurons display a depolarized resting membrane potential and increased resistance, but unchanged other parameters of interest. **A** Immunofluorescence staining of human iPSC-derived cells for β-tubulin III (red), GFAP (green), and nuclear marker Hoechst 33342 (blue) at 130 DIV in a control cell culture (wt tau, left) and a genetically engineered one (right image). Right, quantification of the relative area of GFAP *versus* β-tubulin III shows a similar proportion of astroglia between the isogenic control and *MAPT* IVS10+16 groups (total *n* = 6 cell cultures *per* group, *p* = 0.702; the two-tailed unpaired *t*-test). **B** Statistics of the resting membrane potential (*V*_rest_) of iPSC-derived neurons in control (wt tau) and *MAPT* IVS10+16 groups during late neurogenesis (~190–220 DIV). ***P < 0.001 (two-tailed unpaired *t*-test). **C** Statistics of the capacitance (*C*_m_) of iPSC-derived neurons in control (wt tau) and *MAPT* IVS10+16 groups. Boxes show median values. Nonparametric Mann–Whitney test indicated. **D** Same as in (**B**), but for the input resistance (*R*_in_). The two-tailed unpaired *t*-test indicated. **E** Same as in (**C**), but for the membrane constant (τ_m_). Boxes show median values. Nonparametric Mann–Whitney test indicated. **F** Left, an overlay of the membrane potential changes in response to a hyperpolarizing current (on the top) in a control (wt tau) neuron (black line) and a *MAPT* IVS10+16 neuron (red line). Right plots, statistics of the *V*_drop_ and the sag ratio of (right) in the wt tau and *MAPT* IVS10+16 groups. The two-tailed unpaired *t*-test indicated. All data are mean with s.e.m, unless indicated. The number of tested cells indicated.

We next examined the properties of genetically engendered neurons with the introduced pathogenic *MAPT* IVS10+16 mutation, hence an increased expression of the 4R tau protein [19]. The *MAPT* IVS10+16 neurons displayed depolarized *V*_rest_ at ~190 DIV compared with the control cohort (−51.8 ± 2.2 mV, *n* = 33 in wt tau neurons *versus* −33.0 ± 4.0 mV, *n* = 21 in *MAPT* IVS10+16 neurons, *p* < 0.001; Fig. 2B). This value was close to that in the cells derived from patient samples, as reported earlier (−39.0 mV in patient 1 and −41.5 mV in patient 2) [17].

To address the possible role of astroglial cells in our experiments, we performed immunostaining combined with quantitative analysis of the relative proportion of astrocytes in isogenic iPSC cultures. Similar to our previous studies [17], we found a notable proportion of astrocytes, both in control and *MAPT* IVS10+16 cultures (Fig. 2A). Importantly, this proportion was similar in the age-matched cell culture group (*n* = 5 wt tau and 6 *MAPT* IVS10+16 cultures, *p* = 0.702; Fig. 2A).

There was no significant difference in the *C*_m_ of iPSC-derived neurons between the mutant and isogenic control groups (median value, 33.4 pF, *n* = 35 in wt tau neurons *versus* 26.9 pF, *n* = 21 in *MAPT* IVS10+16 neurons, *p* = 0.279; Mann–Whitney test; Fig. 2C). The τ_m_ values were not different either (median value, 20.8 ms, *n* = 35 in wt tau neurons *versus* 34.0, *n* = 21 in *MAPT* IVS10+16 neurons, *p* = 0.250; Fig. 2E). The similarity of *C*_m_ (a parameter that reflects morphological development) and τ_m_ (indicating biophysical development of the membrane) between the groups effectively rules out compromised maturation of *MAPT* IVS10+16 neurons due to the pathogenic IVS10+16 insertion.

In addition to the depolarized *V*_rest_, *MAPT* IVS10+16 neurons had a significantly increased *R*_in_ (608.1 ± 41.0 MΩ, *n* = 35 in wt tau *versus* 981.0 ± 98.0 MΩ, *n* = 21 in *MAPT* IVS10+16 neurons, *p* < 0.001; Fig. 2D). Again, the value in genetically engineered cells was similar to the increased *R*_in_ of matured iPSC-derived neurons from FTDP-17 patient samples (1000.6 ± 112.7 MΩ from [17]). In an attempt to identify ion channels contributing to the differences in passive membrane properties in *MAPT* IVS10+16 neurons, we recorded *I*_h_, a nonspecific cation current (mixed Na^+^-K^+^ conductance), which largely contributes to setting neuronal *V*_rest_ and *R*_in_ [29], with a greater effect in human than in mouse pyramidal neurons [27]. In full agreement with our previous findings obtained in the neurons derived from patient samples [17], the iPSC-derived neurons displayed *I*_h_ of a similar magnitude, for both control (wt tau) and *MAPT* IVS10+16 groups (Fig. 2F): *V*_drop_ was –143.5 ± 10.8 mV (*n* = 30) in control and –146.7 ± 17.8 mV in *MAPT* IVS10+16 neurons (*n* = 26, *p* = 0.879); the sag ratio was 0.38 ± 0.06 (*n* = 30) and 0.30 ± 0.03 (*n* = 20, *p* = 0.239), respectively. These data thus point to other than HCN voltage-gated channels that might affect the intrinsic excitability of the cells with the pathogenic *MAPT* mutation.

### 4R tau decreases Na^+^- and K^+^-current density, changes channel property of fast-inactivating Na_v_, and reduces Na_v_1.6 protein level

**Fig. 3:**
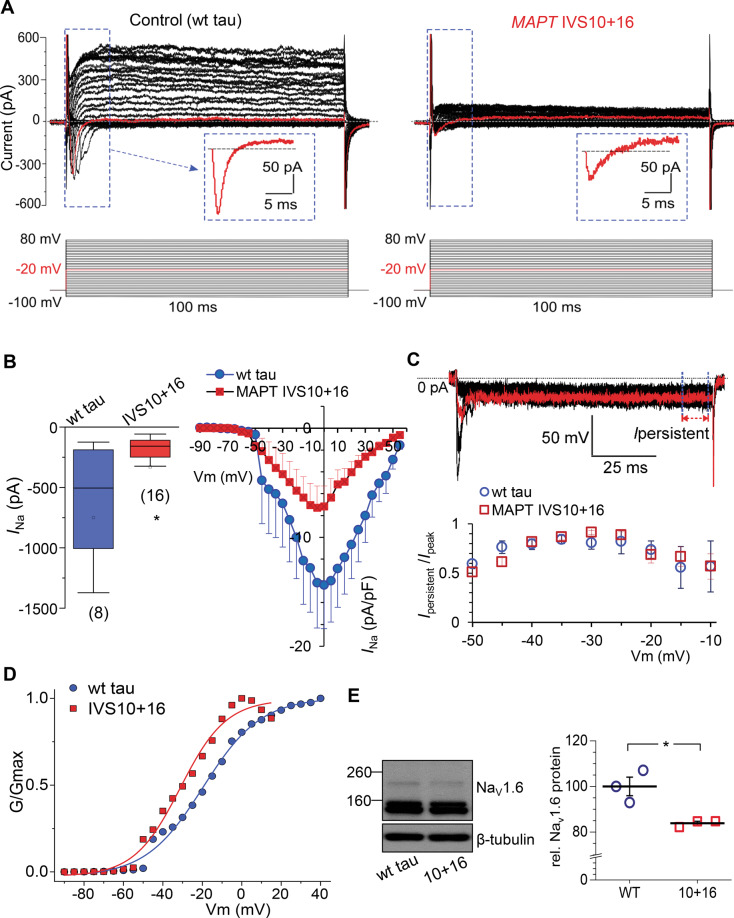
Pathogenic 4R tau decreases both voltage-gated Na^+^- and K^+^-currents, changes Na^+^-channel property, and reduces Na_v_1.6 protein level. **A** Representative patch-clamp recordings showing inward and outward currents recorded from a control (wild type, wt, tau) iPSC-derived neuron (left) and a *MAPT* IVS10+16 neuron (right). The protocol consisted of voltage steps from −100 mV to +80 mV, increment of 5 mV (indicated on the bottom). Red line, the current recorded at –10 mV; box depicts the fast-activating and -inactivating current shown on an expanded scale. **B** Left, statistics for the peak Na^+^-current (*I*_Na_) in isogenic control (wt tau) and *MAPT* IVS10+16 neurons. Boxes show median values; *p < 0.05 (nonparametric Mann–Whitney test). Right, current–voltage (*I–V*) relationship for the *I*_Na_ recorded from both groups of neurons. Protocol as in (**A**). **C** Example of persistent inward current recordings from a *MAPT* IVS10+16 neuron (voltage steps from −50 mV to −20 mV, increment of 5 mV). Red line, the current recorded at –30 mV; arrow and dashed lines depict persistent currents (*I*_persistent_). Statistics of the relative persistent current normalized to peak current for each individual cell in wt tau (*n* = 8 cells) and *MAPT* IVS10+16 groups (*n* = 12 neurons) at membrane potentials tested. **D** Conductance–voltage relationships of *I*_Na_ in wt tau and *MAPT* IVS10+16 neurons. Conductance was normalized to the maximal *I*_Na_ conductance; lines are Boltzmann fitting. **E** A representative western blot for the Na_v_1.6 channel isoform in iPSC-derived neurons at ~200 DIV in wt tau and *MAPT* IVS10+16 cultures with β-tubulin III as loading control (left), and statistics for the protein expression level of Na_v_1.6 channel in both groups (*n* = 3 samples *per* group). *P = 0.018 (the two-tailed unpaired *t*-test). All data are mean with s.e.m, unless indicated. The number of tested cells indicated.

In FTDP-17, human cells derived from patient samples exhibited a reduced current density for the voltage-gated Na_v_ channels [17]. Because the Na_v_ channel superfamily represents one of the key players in neuronal excitability, we asked whether the pathogenic IVS10+16 insertion would lead to the dysfunction of Na_v_ in human iPSC-derived neurons at late neurogenesis. We, therefore, recorded macroscopic voltage-gated *I*_Na_ and *I*_K_ in control (wt tau) and *MAPT* IVS10+16 neurons at different membrane potentials (Fig. 3A).

Our patch-clamp recordings showed that the *I*_Na_ was dramatically reduced, either in its peak amplitude or in current density (normalized by cell capacitance), in *MAPT* IVS10+16 neurons. The median values of current amplitude were –505 pA (*n* = 8) in wt tau neurons, but –158 pA in *MAPT* IVS10+16 neurons (*n* = 16, *p* < 0.05 Mann–Whitney test; Fig. 3B). The *I–V* curves showed a reduced current density in mutant cells, at membrane potentials above approximately –50 mV (Fig. 3B). To understand whether the mutation-induced current reduction relates to a reduced persistent current fraction, we measured the amplitude of the remaining inward current (non-inactivating at the end of a 100-ms pulse, Fig. 3C top) at membrane potentials between –50 mV and –10 mV for each cell. Persistent current, mediated by low-threshold Na_v_1.9 channels, was found in pyramidal neurons of cortical layers over development [30], with its activating threshold near −50 mV [31]. Neither the absolute amplitude nor the relative persistent current fraction (Fig. 3C) in human cells was significantly different between the wt tau (*n* = 8) and *MAPT* IVS10+16 neurons (*n* = 14, *p* > 0.05), at all membrane potentials tested. Thus, the 4R-triggered reduction of *I*_Na_ must be due to a decrease in the fast-inactivating current fraction rather than in the persistent current. We also calculated the conductance for the fast-inactivating current and found a marked difference in the voltage dependence of *I*_Na_ conductance between wt tau and *MAPT* IVS10+16 neurons. The conductance–voltage relationships for *I*_Na_ recorded in both groups showed a shift of the normalized conductance curve in *MAPT* IVS10+16 neurons to more hyperpolarized membrane potentials (Fig. 3D). The voltage at which the channels exhibited half-maximal conductance was −19.60 ± 0.93 mV in the wt tau neurons, but −31.20 ± 1.43 mV in the *MAPT* IVS10+16 neurons (the slope factor, 14.65 ± 0.92 and 11.65 ± 1.39, respectively). These results indicate that 4R tau reduces *I*_Na_ density and changes the fast-inactivating Na_v_ channel properties.

Western blot experiments have further identified the reduced expression of Na_v_1.6 in *MAPT* IVS10+16 neurons at ~200 DIV (*n* = 3 independent samples in wt tau and genetically engineered groups, *p* < 0.05; Fig. 3E). The Na_v_1.6 channel subtype, one of the most abundant neuronal isoforms of the Na_v_ superfamily, largely contributes to the AP initiation and propagation [32]. The present datasets provide further evidence for the mutation-related loss of Na_v_1.6 channel subtype in human cells derived from FTDP-17 patient samples as we found earlier [17].

**Fig. 4:**
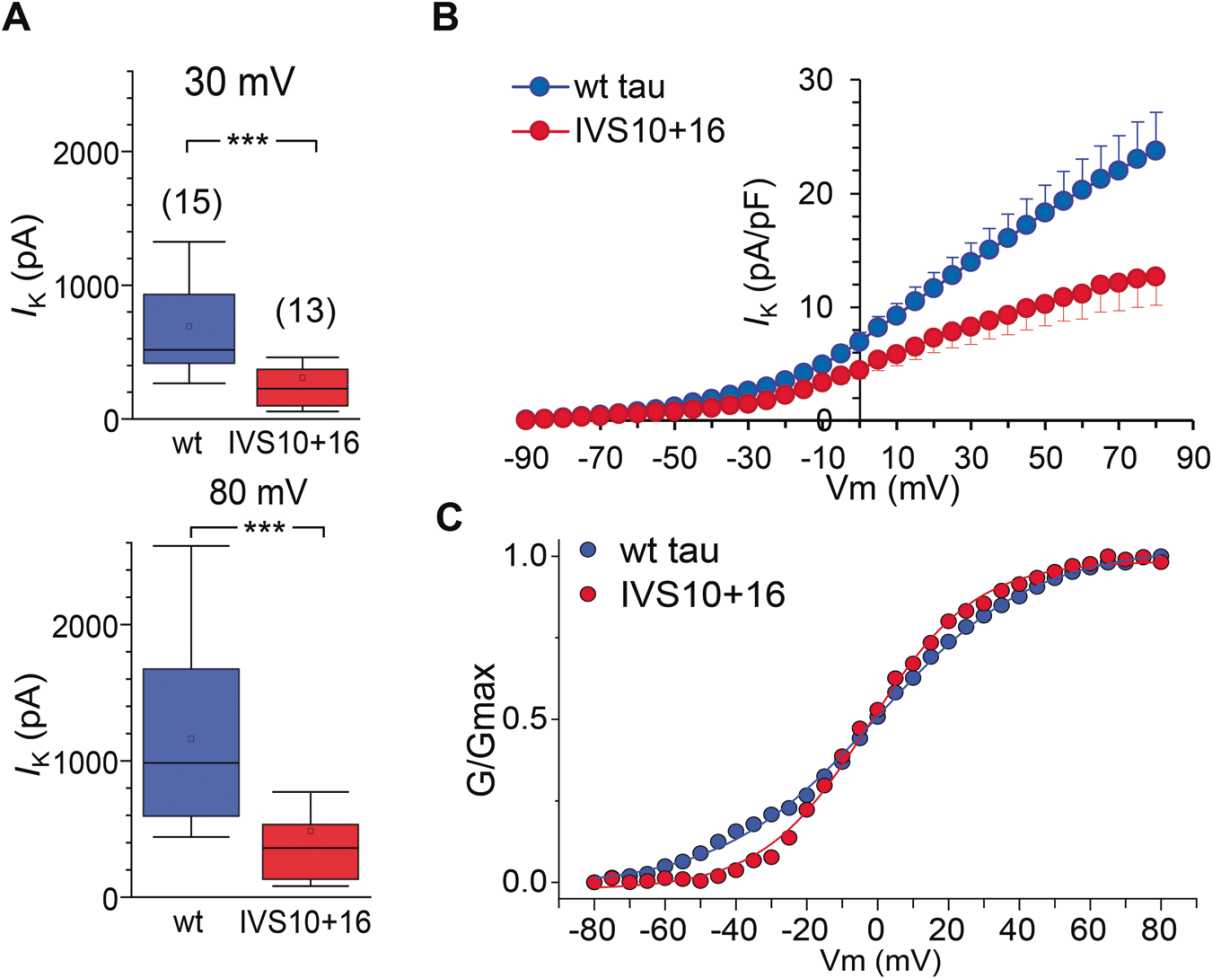
Overexpression of 4R tau decreases the voltage-gated K^+^-current density but not conductance. **A** Statistics for the amplitude of K^+^-current (*I*_K_) measured at 30 mV (upper bars) and 80 mV (lower bars) in isogenic control (wt tau) and *MAPT* IVS10+16 neurons. Boxes show median values; ***p < 0.001 (nonparametric Mann–Whitney test). The number of tested cells indicated. *B. I–V* relationship for the *I*_K_ density in the isogenic control (wt tau) and the *MAPT* IVS10+16 cohorts. C. Conductance–voltage relationships of *I*_K_ recorded in wt tau and *MAPT* IVS10+16 neurons. Conductance was normalized to the maximal *I*_K_ conductance; lines are Boltzmann fitting.

In addition to the changes in *I*_Na_, there was also a robust decrease in *I*_K_ in *MAPT* IVS10+16 neurons compared with the control cohort. We measured macroscopic outward currents at the end of the step pulse, consisting of fast and slowly inactivating *I*_K_ (Fig. 3A), which was significantly diminished (more than halved) in *MAPT* IVS10+16 neurons, either in the amplitude or in the current density. The median values were 516.9 pA (*n* = 13) in wt tau neurons *versus* 226.4 pA (*n* = 15, *p* < 0.001) in *MAPT* IVS10+16 neurons at 30 mV and 984.7 pA *versus* 360.6 pA at 80 mV, respectively (*p* < 0.001 Mann–Whitney test; Fig. 4A). The *I–V* curves showed a dramatically reduced *I*_K_ density at all membrane potentials above 10 mV (Fig. 4B). Notably, the *MAPT* 10+16 mutation had little effect on the conductance–voltage relationship of *I*_K_ (the midpoint was −0.14 ± 0.56 mV in wt tau and −2.58 ± 0.58 mV in *MAPT* IVS10+16 neurons; Fig. 4C). This suggests that 4R tau reduces *I*_k_ density but not its channel properties per se.

### Impaired firing by *MAPT* IVS10+16 neurons and suppressed Ca^2+^ dynamics in cellular compartments during depolarization

Na_v_ and K_v_ channel functions determine neuronal firing activity. While Na_v_ channels are mainly responsible for the initiation of AP generation and the rising phase of AP spike, functional K_v_ channels shape the AP and act to reset the *V*_rest_. Therefore, the 4R tau-triggered decrease in either *I*_Na_ or *I*_K_ should lead to impaired neuronal firing. To assess the anticipated dysfunction, we first carried out whole-cell recordings from *MAPT* IVS10+16 neurons (in current-clamp mode) for the cell firing capacity and second, performed multiphoton excitation fluorescent imaging to monitor the intracellular Ca^2+^ dynamics in cellular compartments during neuronal depolarization.

**Fig. 5:**
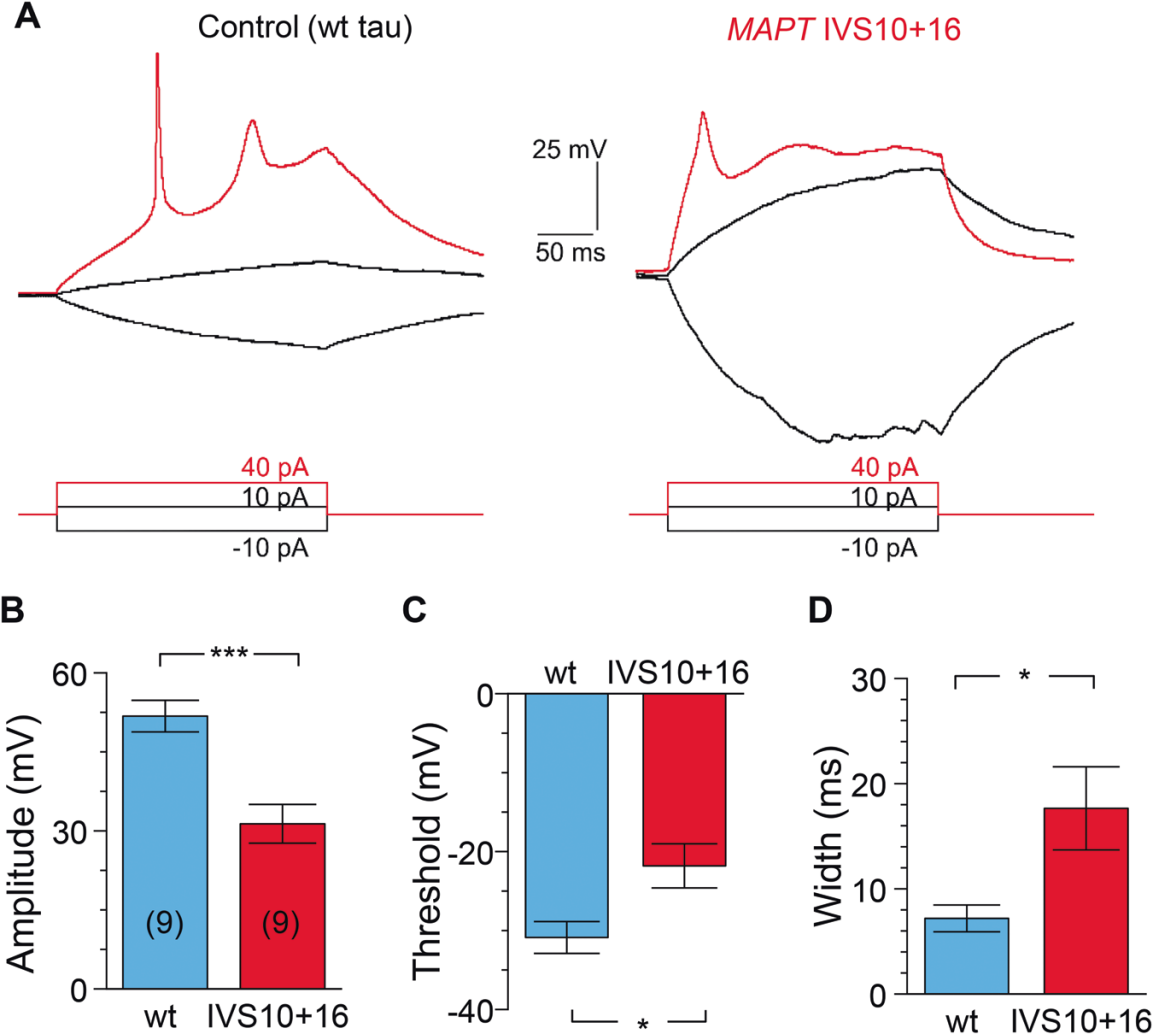
*MAPT* IVS10+16 neurons display impaired action potentials. **A** Example recordings of action potentials (AP) in a control (wild type, wt, tau) iPSC-derived neuron (left) and a *MAPT* IVS10+16 neuron (right) elicited by square depolarizing current pulses (indicated on the bottom). **B**–**D** Statistics for the AP amplitude (**B**), the threshold (**C**) and the width measured at half-maximal amplitude in control (wt tau) and *MAPT* IVS10+16 neurons. Analysis performed for the first AP spike. Data are mean ± s.e.m. ***P < 0.001, *p* = 0.031 for (**C**), and *p* = 0.039 for (**D**) (the two-tailed unpaired *t*-test). Number of cells, *n* = 9 per group.

Electrophysiology revealed an impaired ability of *MAPT* IVS10+16 neurons to fire APs (Fig. 5A). All AP parameters that we tested were severely distorted in *MAPT* IVS10+16 neurons compared with the age-matched control, resulting in a dramatically changed AP waveform. Firstly, the AP spike was reduced (a drop in the amplitude by ~50%, *p* < 0.001; Fig. 5B). Secondly, the AP threshold was more depolarized compared with that in isogenic control (a shift in ~9 mV, *p* < 0.05; Fig. 5C). Thirdly, the spike was substantially wider (>2.5-fold increase in the half-amplitude width, *p* < 0.05; Fig. 5D). These data, again, recapitulate the pathophysiological AP waveform observed earlier in cells derived from FTDP-17 patient samples [17].

**Fig. 6:**
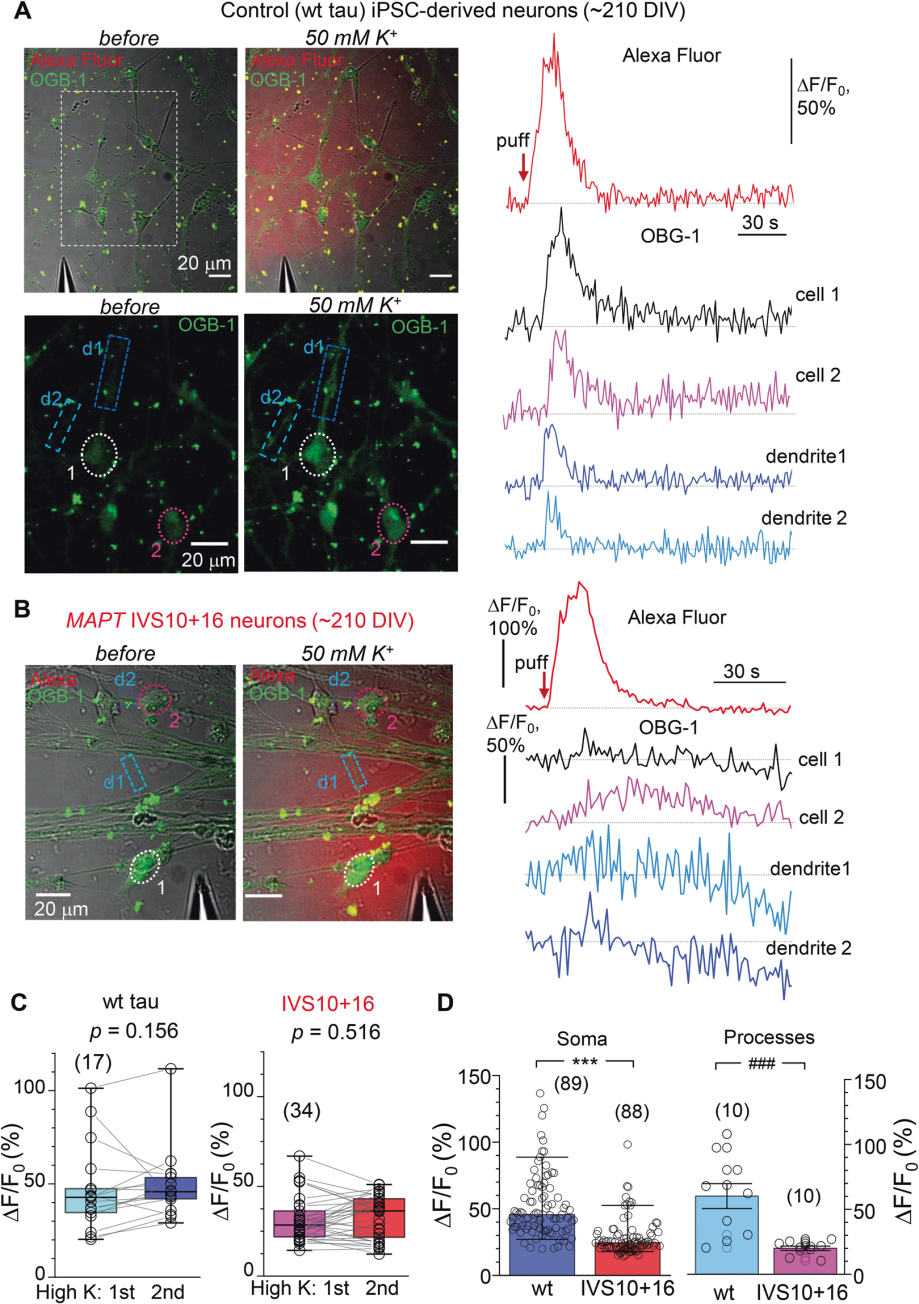
4R tau impairs the intracellular Ca^2+^ mobilization across neuronal compartments upon depolarization. **A** Monitoring of depolarization-induced intracellular Ca^2+^ dynamics using two-photon excitation time-lapse imaging in control (wild type, wt, tau) iPSC-derived neurons (~210 DIV). Images, experimental arrangement for focal application of a high-potassium medium (50 mM) through a micropipette positioned in close proximity of the cells before (left panel) and during a puff of K^+^ (right panel). Images, combined transmitted light, OGB-1 (green), and Alexa Fluor-594 (red) channels (upper row) and OGB-1 only (lower row); *λ*^2P^_ex_ = 800 nm; dotted areas, regions of interest (ROI): cells 1–2 and two neurites, d1 and d2. Plots, the time course of the intracellular Ca^2+^ rise (Δ*F*/*F*_0_ OGB-1 signal) within indicated ROIs before and following cell depolarization; top trace, Alexa Fluor-594 diffusion profile across the field of view; red arrow, timestamp of K^+^ puff. **B** Notations as in (**A**) but for the *MAPT* IVS10+16 group. Note the slow and diminished depolarization-induced Ca^2+^-rise (Δ*F*/*F*_0_ OGB-1 signal), with prolonged decay kinetics, in soma and neurites. **C** Quantification of the peak Ca^2+^-rise (Δ*F*/*F*_0_ OGB-1 signal) in response to the two consecutive applications of K^+^ medium in individual neurons from control (wt tau, *n* = 17 cells, left bars) and *MAPT* IVS10+16 groups (*n* = 34, right). Boxes show median values. The nonparametric paired-sample Wilcoxon signed rank test indicated. **D** Statistics for the depolarization-induced Ca^2+^-rise (Δ*F*/*F*_0_ OGB-1 signal) for pooled groups of wt tau and *MAPT* IVS10+16 neurons recorded in soma (bars show median values, *n* = 89 cells and 88 cells, respectively, three independent preparations) and within neuronal processes (mean ± SEM, *n* = 10 *per* group). ***p < 0.001 nonparametric Mann–Whitney test; ^###^*p* < 0.001 ANOVA test.

Multiphoton fluorescent imaging has confirmed impairments in *MAPT* IVS10+16 neurons during depolarization. Using a highly sensitive Ca^2+^ indicator OGB-1, we detected an intracellular Ca^2+^ rise in control neurons (~210 DIV) in response to a brief puff of high-potassium solution (Fig. 6A). This experimental design provided a very mild stimulus (arguably ‘physiologically compatible’) rather than a commonly used bath agonist application that is typically associated with profound neuronal hyper-excitation across cell culture. A bright morphological tracer Alexa Fluor 594 (AF) inside a puff-pipette was used to monitor the spatiotemporal profile of the ejected medium (Fig. 6A, B left panel). The depolarization-induced Ca^2+^ rise (the Δ*F*/*F*_0_ OGB-1 signal amplitude) was transient, fully consistent with the time course of the AF signal profile. We detected a fast Ca^2+^ rise in both somata and neurites, which returned to the baseline level, indicating physiological compatibility of the intracellular Ca^2+^ dynamics in control (wt tau) neurons (Fig. 6A right panel). The response was consistent and reproducible, showing a similar amplitude of Ca^2+^ rise between trials in isogenic control (*p* = 0.156, paired nonparametric Wilcoxon test) or mutant cells (*p* = 0.516, paired nonparametric Wilcoxon test; Fig. 6C). The median amplitude of the depolarization-induced Ca^2+^-sensitive OGB-1 signal was 45.6% Δ*F*/*F*_0_ in control neurons (at least 89 cells, three independent samples; Fig. 6D); however, it was almost halved in *MAPT* IVS10+16 neurons: median value, 24.4% (*n* = 88 neurons, *p* < 0.001 compared with the wt tau; Fig. 6D). We rarely observed an occurrence of the depolarization-induced Ca^2+^ rise in neurites in *MAPT* IVS10+16 neurons, which amplitude was also dramatically reduced (approximately three times): mean, 60.2 ± 9.6% in control versus 20.0 ± 1.7% in *MAPT* IVS10+16 neurons (*n* = 10 *per* group, *p* < 0.001 ANOVA test; Fig. 6D).

## Discussion

This study provides an electrophysiological characterisation of human cells with the genetically engineered pathogenic IVS10+16 monoallelic mutation, which was introduced into the healthy donor cells to increase splicing of the 4R tau protein isoform. Our data document the pathological phenotype of intrinsic excitability of the generated neurons and provide the first experimental evidence for the 4R tau-induced impairments in the biophysical properties and neuronal activity of human cells. The findings are consistent with the earlier described phenotype of pathophysiological excitability of the cells derived from FTDP-17 patient samples, thereby confirming that overproduction of 4R tau by introducing the *MAPT* 10+16 mutation into healthy cells effectively reproduces the pathogenesis of neurons derived from patient samples.

As noted in the introduction, deposition of 4R tau aggregates is associated with a range of neurodegenerative disorders, such as progressive supranuclear palsy, corticobasal degeneration, FTDP-17, argyrophilic grain dementia, globular glial tauopathy, ageing-related tau astrogliopathy, others [33–36]. Post-mortem studies have revealed tau aggregations throughout brain regions in a form of dense and prominent plagues, either neuronal or glial, distributed in equal measure in astrocytic processes and oligodendroglia [37–39]. After years of studying clinical cases and designing experimental models of tau pathology, more than 25 tau mutations have been identified to date [40], although the vast majority of the mutations remains unattributed to the known tau-related neurodegeneration causing dementia. Because the expression of 4R tau protein is determined by alternative splicing of exon 10 [1], mutations promoting the inclusion of exon 10 lead to the overproduction of 4R tau, hence the 4R tau-related neuropathology. As a strategy to explore the link between the 4R tau and human neuropathology, in the present study we have exploited the advances of engineering the 4R tau-induced mutations and modelling human dementia in vitro.

Genetically engineered neurons were human iPSC-derived neurons with the *MAPT* IVS10+16^+/−^ mutation introduced into the healthy donor cells, which showed an increased expression of the 4R tau protein associated with tau hyperphosphorylation at different phosphorylation sites (Ser396/Ser404 and Thr181) [19]. However, spontaneous formation of tau aggregates was not found in iPSC cultures [41], leading to a common assumption that endogenous tau is not sufficient to trigger the formation of tau aggregates in iPSC-derived neurons. Aggregation of tau protein was observed in other models of tauopathies exerted by introducing a combination of several mutations [42] or in conjunction with recombinant tau, K18 [43].

Our electrophysiological investigation of iPSC-derived neurons shows that the *MAPT* IVS10+16^+/−^ mutation leads to severe neuronal dysfunction. Overall, it was evidenced by (i) a significantly depolarized *V*_rest_ and (ii) an increased *R*_in_ at late neurogenesis, (iii) decreased functional Na_v_ (fast-inactivating) and iv) K_v_ channels that ultimately led to v) an impaired firing activity of *MAPT* IVS10+16 neurons compared with the age-matched isogenic control (parental cell lines with wt tau). Importantly, genetically engineered neurons recapitulated the neuropathology of human cells derived from FTDP-17 patient samples, across all parameters of interest [17]. The phenotypic profile of *MAPT* IVS10+16 neurons could not be explained by a developmental delay due to the introduced mutation. Some of the key properties of genetically engineered neurons were similar to those in the age-matched control group, such as cell capacitance (a parameter of morphological development), the sag ratio and *V*_drop_ (either of two related to the HCN channel function). In addition to electrophysiological comparisons, we routinely observed a significant proportion of astroglial cells in iPSC cultures over late neurogenesis, as noted in our earlier study [17] and here (Fig. 2A). The proportion of astroglia remained similar in control and mutant cohorts, providing an additional line of evidence that the pathophysiology of genetically engineered neurons relates not to cell quality nor other detrimental effects of long-term iPSC culture maintenance.

Intrinsic membrane excitability determines neuronal activity, in particular, the cell firing capacity. This primary neuronal function was severely impaired in *MAPT* IVS10+16 neurons. The waveform of AP was dramatically changed, including a reduced amplitude, a widened spike, and a depolarizing shift in the threshold (by ~9 mV)—these effects were similar to what we have observed in iPSC-derived neurons from FTDP-17 patient samples [17]. The reduced firing capacity of neocortical neurons was reported in different transgenic models of tauopathy [44–46]. Our voltage-clamp recordings reveal the 4R tau-induced decrease in macroscopic Na^+^- and K^+^-currents, indicating downregulated functional Na_v_ and K_v_, the two key ion channel families responsible for neuronal firing. These data might explain a biophysical basis of aberrant neuronal activity in dementia. Further to this, it appears that 4R tau changes channel properties of fast-inactivating Na_v_ (half-maximal channel conductance shifted by ~11 mV to more hyperpolarized voltage). A mechanistic basis of the tau-channel interaction requires dedicated investigations at the molecular level: several studies have provided some insights into the tau–membrane interaction, demonstrating increased binding of the hyperphosphorylated tau into bilayer membranes to form a membrane-mediated tau aggregation [47, 48] associated with membrane invagination and rapid membrane destabilization [49]. This effect would suggest an impaired ion channel function and distorted channel/receptor trafficking [50], which are adversely affected by the hyperphosphorylated tau interfering with ribosomal protein synthesis and scaffolding [51]. In this context, the present and a related earlier study [17] have extended our understanding of the impaired neuronal firing by identifying the loss of Na_v_1.6 channel subtype triggered by the 4R tau isoform. The Na_v_1.6 channel is broadly distributed and highly expressed, both in neurons and glia [52]; its altered function was also found contributing to neurological and psychiatric brain disorders, i.e. epileptic encephalopathy, ataxia, dystonia, others [53–55].

Live-cell multiphoton excitation imaging used here unveiled the 4R tau-induced impairments in intracellular Ca^2+^ handling in human iPSC-derived neurons during cell depolarization. The intracellular Ca^2+^ mobilization was perturbed across cellular compartments, in somata and neurites, upon a mild, ‘physiologically compatible’, depolarizing stimulus (a brief local puff of high potassium solution). This approach contrasts with the majority of published studies in the field, in which agonist(s) are commonly bath applied, producing an enormous, arguably non-physiological, cell activation, triggering thus an intracellular Ca^2+^ rise that commonly exceeds neuronal Ca^2+^ signals reported in experiments in vivo. The Ca^2+^-based concept of neurodegeneration has been suggested as a primary mechanism underpinning the pathogenesis in Alzheimer’s disease, in particular, due to an increased Ca^2+^ influx causing Ca^2+^-induced neuronal excitotoxicity [56] and astroglial Ca^2+^ hyperactivity [57, 58].

Evidence for the causal connection between human tau isoforms and Ca^2+^ signalling impairments remains sparse, let alone studies in human cells. The pathogenic tau protein was found to promote Ca^2+^ influx through muscarinic receptors [59] and glutamate receptors in primary brain neurons and astrocytes[12, 60], but failed to induce Ca^2+^ release from Ca^2+^ stores of the endoplasmic reticulum [10]. It was also reported that the pathogenic tau triggers depletion of nuclear Ca^2+^ in human iPSC-derived neurons, driving cell death [11]. This is in line with our recent observations of the absent glutamate-induced intracellular Ca^2+^ rise in the human iPSC-derived neurons with pathogenic 4R tau stimulated in a calcium-free medium [12]. Notably, tau inhibited Ca^2+^ efflux by the mitochondria in both primary and human iPSC-derived neurons with the *MAPT* 10+16 mutation [60]. Taken together, these data appear to show that [10] the extracellular Ca^2+^ entry is the primary source of intracellular Ca^2+^ mobilization during cells’ stimulation. Some significant uncertainty remains regarding the 3R/4R tau-induced changes in the intracellular Ca^2+^ mobilization at various time points, i.e. the earlier appearance of pathogenic tau *versus* the overt stages of tau-induced neuropathology. A very recently discovered mechanism of aberrant neuronal excitability and Ca^2+^-induced cell death in FTDP-17 [12] includes the tau-induced overproduction of reactive oxygen species (ROS) by mitochondria. The latter causes heavy oxidation of intracellular proteins, resulting in altered expression of AMPA and NMDA receptor subunits and augmented Ca^2+^ influx through these receptors. Whether the likely ROS-produced damage to other membrane ion channels that dictate neuronal excitability (Na_v_ and K_v_ superfamilies, Ca^2+^ channel family, others) contributes to the neuropathological excitability of human cells in the 4R tau pathology remains to be further elucidated.

## Data Availability

The datasets generated and analysed during this study are included in this article.
